# Submassive Pulmonary Embolism Treated With Catheter-Directed Thrombolysis in Resource-Limited Setting: A Case Report and Review of Literature

**DOI:** 10.7759/cureus.21760

**Published:** 2022-01-31

**Authors:** Hitesh Gurjar, Himani Singh, Barkha Gurjar

**Affiliations:** 1 Internal Medicine, BronxCare Health System, Icahn School of Medicine at Mount Sinai, Bronx, USA; 2 Department of Radiology, Ivy Hospital, Nawanshahr, IND; 3 Department of Obstetrics and Gynecology, Jawaharlal Nehru Medical College, Ajmer, IND

**Keywords:** resource-limited setting, catheter-directed thrombolysis, submassive pulmonary embolism, alteplase, endovascular catheter-directed thrombolysis

## Abstract

We describe a case of a young male who presented with acute onset progressively worsening shortness of breath for four days duration. He used to smoke cigarettes, and his profession required prolonged periods of standing. He underwent a two-dimensional echocardiogram showing right ventricular (RV) strain and computed tomography (CT) showing thrombus in the left major pulmonary artery. His pulmonary embolism severity index (PESI) score was high, predicting higher short-term mortality. Treatment options including risks and benefits were discussed with the patient, and he underwent catheter-directed thrombolysis (CDT) with rapid resolution of symptoms, oxygen saturation, and pulmonary artery pressures (PAP). He was discharged home safely after successful treatment of his condition.

## Introduction

Pulmonary embolism (PE) remains an underdiagnosed and life-threatening clinical condition [1]. Massive PE is usually treated with 100 mg of alteplase infusion over two hours [2]. However, the treatment options for submassive PE are not well established in spite of significant mortality in this clinical condition. Lately, there has been increasing use of pulmonary embolism response teams (PERT) and catheter-directed interventions (CDI), including ultrasound-guided thrombolysis, thrombectomy with thrombus aspiration catheters, and catheter-directed delivery of lower dose thrombolytic agents [3]. We present a case of a young male with submassive PE treated with catheter-directed thrombolysis (CDT) with alteplase.

## Case presentation

A 33-year-old male presented to our hospital with complaints of new-onset exertional breathlessness for four days. He did not have any significant past medical history. He used to smoke cigarettes and worked at a salon requiring prolonged periods of standing. Four days before the presentation, he started feeling an increasing level of breathlessness, which started suddenly and has progressed over four days to a level where he started feeling breathless even on minimal activities of daily living.

In the emergency department, the patient appeared well and was not in distress. He was afebrile with a temperature of 97.8°F; his blood pressure was 96/58 mmHg, heart rate was 116 beats per minute, respiratory rate was 22 breaths per minute, and oxygen saturation was 88% while breathing ambient air. Cardiovascular examination was within normal limits, except for the tachycardia.

His initial laboratory findings were significant for a white blood cell count of 11,700 per cubic millimeter (reference range: 4,000-11,000 cubic millimeter), and his qualitative troponin levels were positive. An electrocardiogram showed sinus tachycardia. Chest X-ray did not show any infiltrates.

Differential diagnosis and decision-making

In a young male patient with a history of smoking, dyspnea should be approached systematically. The causes can be divided into systemic factors or organ-specific. Among systemic causes, anemia is the prime consideration, with many patients with acute blood loss anemia presenting as dyspnea, syncope or fatigue, and generalized weakness, even in the absence of overt bleeding history, and should always be kept as one of the differentials.

Cardiac causes can be divided into pericardial, myocardial, endocardial, valvular, and vascular. Pericardial causes such as pericarditis and pericardial effusion are excluded by the absence of a history of pleuritic chest pain, absence of pericardial rub, raised jugular venous pressure, muffled heart sounds on examination, and absence of electrocardiographic or roentgenographic changes. Similarly, myocardial causes such as myocardial infarction or cardiomyopathy such as dilated or restrictive cardiomyopathy can be excluded by the combination of history, physical examination, and ECG and echocardiogram. Endocardial causes such as endomyocardial fibroelastosis are exceedingly rare. Vascular conditions such as coronary artery thrombotic complications, spontaneous coronary artery dissections, or other coronary artery anomalies are diagnosed based on angiograms once other common conditions are excluded.

Similarly, lung conditions can be approached as pleural-based conditions presenting as pleuritic chest pain and asymmetric lung examination findings. Parenchymal conditions are usually accompanied by sputum production and hemoptysis, airway conditions present as wheezing and bronchospasm or stridor, and mediastinal conditions are usually accompanied by dysphagia and X-ray evidence of mediastinal widening. Vascular lung conditions are an important consideration in patients with dyspnea, hypoxia, and tachycardia. Pulmonary embolism is an often missed diagnosis in the absence of a history of prolonged immobilization. A high degree of suspicion, even in ambulatory patients, helps in the correct and timely diagnosis of this condition.

Further evaluation

A two-dimensional echocardiogram was done, and it showed dilatation of the right atrium and right ventricle (RV) with estimated pulmonary artery pressures (PAP) of 55 mmHg, with evidence of free wall hypokinesia. Computed tomography (CT) angiogram of the chest was done, which showed a thrombus in the left major pulmonary artery branches. In view of a high pulmonary embolism severity index (PESI) score of 113, he was admitted for stabilization and treatment. After shared decision-making with the patient and lengthy discussion regarding various treatment options, catheter-directed thrombolysis was opted as the treatment option.

Treatment and procedural details

**Figure 1:**
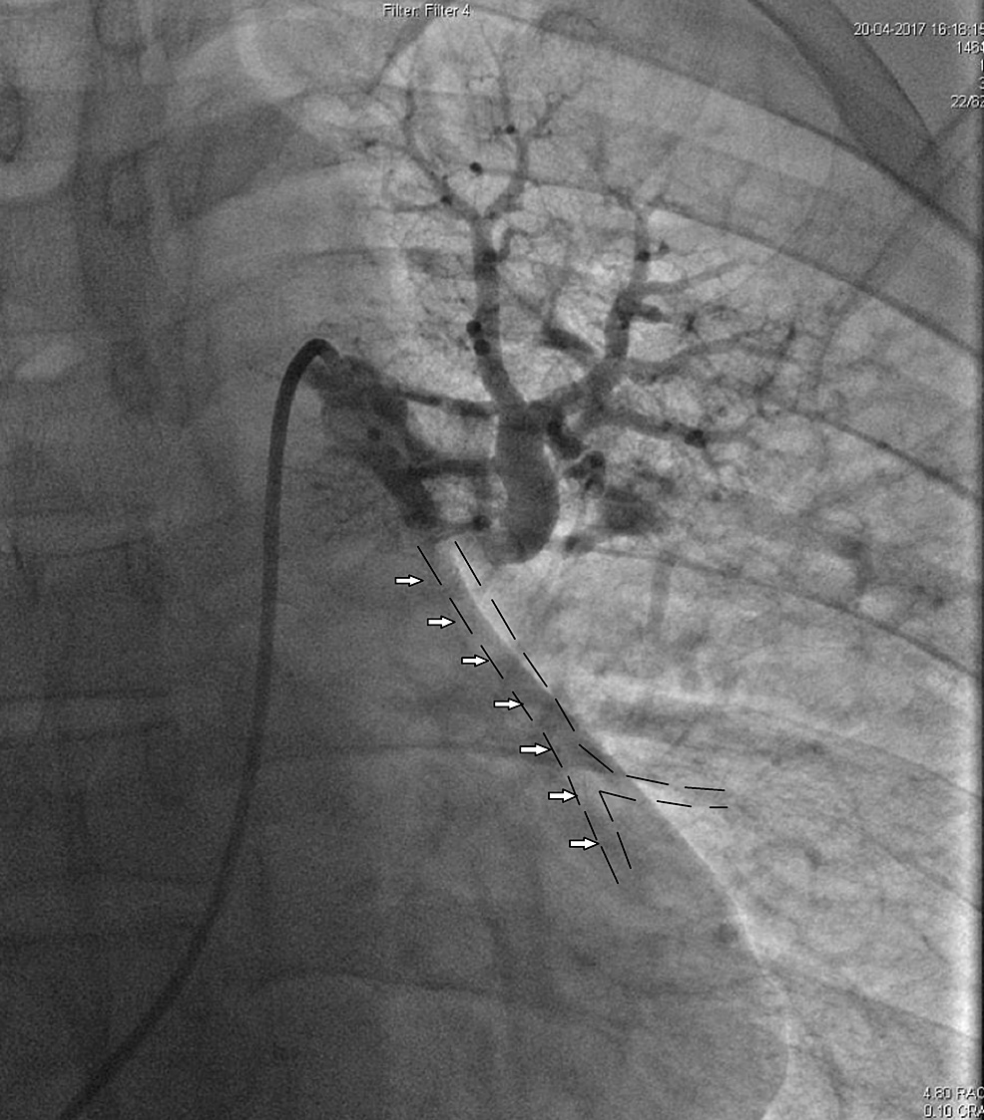
Injection via 6 Fr JR 3.5 guiding catheter into the left pulmonary artery showing the total cutoff of the left interlobar artery (arrows and broken lines showing anticipated artery, which is non-opacified with the contrast due to the thrombus) JR: Judkins right

On presentation, the patient was started on unfractionated heparin and 0.9% sodium chloride infusion. He was admitted to the intensive care unit and taken to the cardiac catheterization suite. The right common femoral vein was punctured and cannulated with a 7 Fr sheath. A simultaneous femoral arterial 6 Fr sheath was placed for invasive hemodynamic monitoring. Initial inferior vena cava (IVC) angiogram was done through a hand injection with iodinated contrast through the femoral venous sheath. This step is important to avoid missing any thrombus in route of catheter advancements and hence avoid catastrophic outcomes where the thrombus present in IVC can be further advanced to the heart and pulmonary circulation. A 7 Fr Judkins right (JR) 3.5 guiding catheter VISTA BRITE TIP® (Cordis Corporation, Miami, Florida, USA) was advanced over a 0.035 inch × 260 cm hydrophilic exchange length Terumo GLIDEWIRE® guidewire (Terumo Corporation, Tokyo, Japan). Once the catheter was advanced through the right atrium, right ventricle, main pulmonary artery, and into the left main pulmonary artery branch, the contrast was injected, and it showed occlusion of the left interlobar pulmonary artery, as shown in Figure 1.

**Figure 2:**
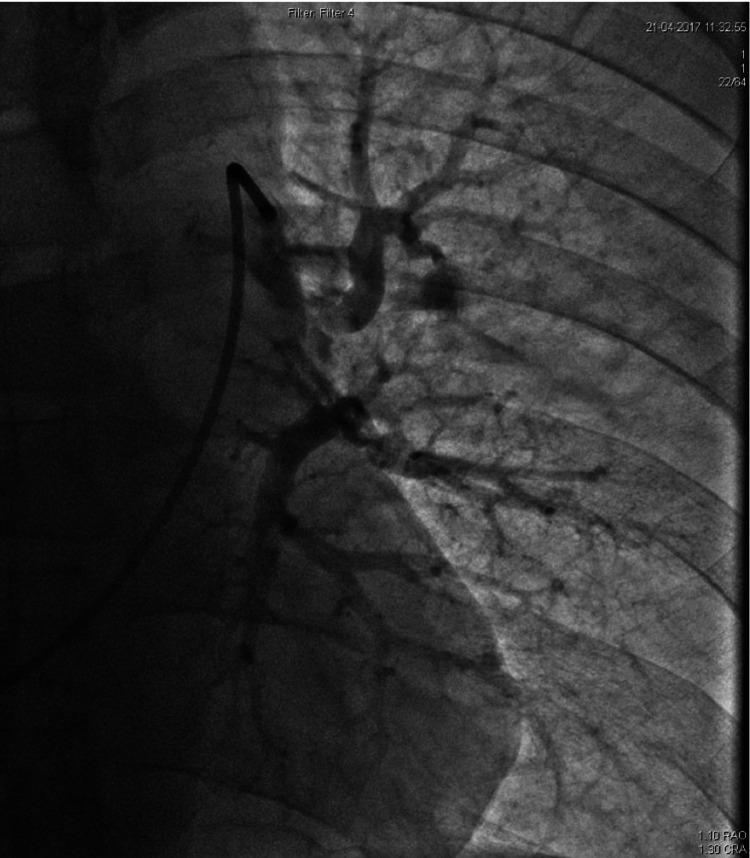
Repeat injection showing the resolution of the thrombus after the 24-hour infusion of alteplase via a catheter

After a 5-mg bolus of alteplase into the thrombotic segment, the catheter was left in situ, and alteplase was started at 0.5 mg per hour infusion through the catheter. Pulmonary artery pressure (PAP) was measured before the alteplase infusion, and it was recorded as 45 mmHg. The patient was monitored continuously in the coronary care unit, and his PAP was also monitored. A repeat angiogram was done after the 24-hour infusion, and it showed significant reduction in thrombus burden, as shown in Figure 2. The catheter was removed over guidewire after a final pulmonary arterial and right ventricular angiogram. This step is important to have a final assessment of the angiographic thrombus burden, as well as identify any mechanical complications.

The patient started having significant symptomatic improvement in dyspnea within four hours of infusion, and his oxygen saturation improved to 95% after six hours of infusion. His PAP at six, 12, and 24 hours were 40, 32, and 22 mmHg, respectively. He was discharged home after a 48-hour stay. Rivaroxaban was started 24 hours after alteplase infusion completion, and he was advised smoking cessation.

Follow-up

He was followed after the procedure in outpatient setting. He was prescribed direct oral anticoagulant (DOAC) rivaroxaban 15 mg two times a day for three weeks, followed by 20 mg once a day. He returned to his work as a hairdresser after a week. He was continued on DOACs for six months, and his follow-up echocardiogram after six months showed normal right atrial and right ventricular size. He has quit cigarette smoking. His effort tolerance continues to be unlimited, and he did not show any signs of chronic thromboembolic pulmonary hypertension.

## Discussion

Pulmonary embolism (PE) is a life-threatening disease with three-month mortality of 15.3% [4]. It is often missed as a diagnosis during the antemortem period, being suspected only in 8%-30% of patients in whom PE contributed to death [1,5]. Since the advent of imaging studies such as high-resolution computed tomography (HRCT), it is diagnosed more often in the current era, but its prevalence in autopsy series has remained fairly constant at around 14.6% over the past many decades [1].

The treatment options for PE are based on clinical categories of massive, submassive, and small PE, as defined by the American Heart Association (AHA) [3]. The European Society of Cardiology (ESC) has further divided submassive PE or the intermediate-risk group into intermediate-high-risk or intermediate-low-risk groups [6]. Treatment options can be grouped into systemic full-dose thrombolysis, systemic low-dose thrombolysis, catheter-directed interventions, anticoagulation alone, and surgical treatment. For massive PE, systemic thrombolysis is usually preferred due to its rapidity of action and risk of rapid clinical deterioration. The recommended dose of alteplase is 100 mg over two hours [2]. Low-dose thrombolysis with alteplase 50 mg have been found to be safe and effective in moderate PE [7]. However, still due to the high dose administered over a short duration, many patients have bleeding complications such as life-threatening internal bleeding. In the submassive PE group, in the International Cooperative Pulmonary Embolism Registry (ICOPER) registry, mortality was 14.7% when thrombolysis was not used; however, it was 21% when patients in this group were treated with thrombolysis [4]. In patients in whom systemic thrombolysis is contraindicated or in whom the risks outweigh the benefits, such as in submassive PE, other approaches may be considered, including surgical thrombectomy or catheter-directed interventions. The treatment options are usually decided after detailed discussion with patients about the risks and benefits, treatment options available, and associated clinical and hemodynamic considerations such as chronic obstructive airway disease or preexisting left or right heart conditions causing congestive heart failure. Since there is clinical equipoise in submassive PE regarding therapeutic options, it is imperative to consider the clinical judgment and individualize the treatment option.

To answer this equipoise in the subset of patients with submassive PE, two large studies were carried out, namely, the Pulmonary EmbolIsm THrOmbolysis (PEITHO) and the Tenecteplase Or Placebo: Cardiopulmonary Outcomes AT three months (TOPCOAT). These studies compared systemic thrombolysis with anticoagulation versus anticoagulation alone and have found less risk of hemodynamic compromise and secondary endpoints at three months, respectively. However, systemic thrombolysis was associated with increased major stroke and hemorrhage [8,9]. Similarly, three studies have evaluated the role of CDT in submassive PE. The Ultrasound-Assisted Catheter-Directed Thrombolysis for Acute Intermediate-Risk Pulmonary Embolism (ULTIMA) trial compared ultrasound-assisted CDT to anticoagulation and showed improved right ventricular (RV) dilatation and dysfunction more quickly [10]. The SEATTLE II study also showed improved hemodynamics and RV dilatation and decreased pulmonary artery pressure [11]. The Pulmonary Embolism Response to Fragmentation, Embolectomy, and Catheter Thrombolysis (PERFECT) study also achieved clinical success without major complications [12]. Similarly, in our patient, we considered CDT after discussion with the patient, and CDT over a duration of 24 hours was delivered. Pigtail catheter to mechanically disrupt the thrombus, ultrasound assistance via EKOS catheter (Boston Scientific Corporation, Marlborough, Massachusetts, USA), and the Venturi effect through saline injection via AngioJet catheter (Boston Scientific Corporation, Marlborough, Massachusetts, USA) have been used to enhance the delivery of thrombolysis at local microenvironment [11,13,14]. The use of any particular technique over simple catheter-directed delivery of thrombolytic agent has not shown any added benefit, and the choice depends on operator preference at this point [15]. Angiographic thrombus burden as assessed by serial imaging has not been associated with adverse clinical outcomes at follow-up [16].

Hence, when the pulmonary embolism severity index (PESI) score is high in patients with submassive PE, CDT can be considered, as this subgroup of patients has higher chances of escalation of therapy, which include initiation of mechanical ventilation and start of vasopressors. If such treatment is not available at a particular center, these cases can be considered for referral to centers capable of transcatheter interventions.

CDT is usually a relatively safe procedure; however, it is associated with a few important side effects, such as hemorrhagic stroke, vascular access site-related complications such as hematoma, retroperitoneal bleeding, catheter-induced trauma to right-sided valvular structures, bradycardia and complete heart block, pulmonary artery dissection, or pericardial effusion [17].

## Conclusions

CDT is currently recommended only for patients with failed thrombolysis, contraindications to thrombolysis, or submassive PE based on individual risk stratification and judgment. However, it offers safe and effective treatment options in the treatment of this important subgroup of patients.
